# Editorial: The challenges of consciousness research in light of the variations of conscious experience

**DOI:** 10.3389/fpsyg.2024.1370419

**Published:** 2024-02-26

**Authors:** Christopher Gutland, Sucharit Katyal, Kelin Li, Marcela Venebra Muñoz, Alexander Nicolai Wendt

**Affiliations:** ^1^School of Philosophy, Zhejiang University, Hangzhou, China; ^2^Max Planck Centre for Computational Psychiatry and Ageing Research, University College London, London, United Kingdom; ^3^School of Philosophy, Renmin University of China, Beijing, China; ^4^Instituto de Investigaciones Filosóficas, Universidad Autónoma del Estado de México, Toluca, Mexico; ^5^Faculty of Psychology, Sigmund Freud University Vienna, Vienna, Austria

**Keywords:** constants in conscious experience, variations of conscious experience, adults, methodology, consciousness research

Conscious experience undergoes considerable changes. Prominent examples are the transitions between dreaming, deep sleep, and the unfolding of consciousness in early childhood. However, the conscious experience of awake adults can change no less drastically. In adults, such changes can occur accompanied by more or less control. Examples of uncontrolled or even uncontrollable shifts are psychotic episodes, developing depression, schizophrenia, or mania. Arguably, examples of more controlled changes are those brought about by deeply immersing oneself in artworks or artistic performances, cultural rituals geared toward a state of trance, and even administering drugs like hallucinogens. The most controlled manner to bring about such changes is through meditative practices and philosophical methods of exploring the constitution of consciousness.

This situation creates challenges for consciousness research: After all, an objective or intersubjective investigation requires observer-independent statements about what conscious experience is like. The named changes, however, mean that the object of consciousness research, i.e., consciousness, is, in a sense, unstable. Conscious experience may vary considerably, even in adults. Three questions in need of answering thus emerge for consciousness research: First, can we identify parameters that help us understand and describe these changes? Second, can we identify aspects, elements, or structures of consciousness that remain constant even within such changes? Third, is it possible to determine an “average” in the sense of an everyday consious experience that could serve as a frame of reference for contrasting the possible changes?

Those are the main questions underlying the contributions to this volume. We limited the investigation to the conscious experience in adults, and we welcomed interdisciplinary contributions combining fields like psychology, psychiatry, philosophy, meditation research, and neuroscience. A single volume naturally cannot reach a conclusive and comprehensive scientific description of all the possible alterations and their mutual relations. However, we are hopeful that the contributions united here will incite awareness of the challenges in describing the dynamics of conscious experience and provide means to tackle them scientifically. In the following brief summary of each article, we proceed in alphabetical order by last name of the author, and we focus on how each contribution relates to the overall goal of this volume.

Christoffersen et al. submit a literature review on the notion of tranquility, focusing on patterns of regional traditions. Providing a system of four experiential categories, the authors engage with the question of whether the localized traditions share phenomenological patterns. In general, most, if not all, experiences of tranquility share the structural character of detachment.

Dzwiza-Ohlsen and Kempermann explore the embodied mind in motion as a framework for understanding dementia from neuroscientific and philosophical perspectives. The authors discuss habits as embodied long-term memories and illustrate this with Marta Cinta González Saldaña, an ex-ballerina with Alzheimer's disease. The example shows that highly habitually embodied abilities are less prone to undergo the change of consciousness occuring in Alzheimer's disease.

Guardiola explores different distinctions within the ego drawn by Edmund Husserl and their explanatory value for depersonalization disorders. He first offers reflections on how Descartes's philosophy unduly suggested identifying the ego and the subject. After laying out three senses of the ego in Husserl—the ego pole, the substrate of habitualities, and the monadic ego—Guardiola then suggests that dislocated mereological relations between the first two senses can explain psychotic or schizophrenic experiences.

Gutland explores a change in conscious experience when transitioning from thinking quantitatively to thinking qualitatively. In the first part, he draws on Edmund Husserl to show how science historically and one-sidedly emphasized quantification and measurability while discarding the objectivity of experiential qualities. Drawing on Hegel, Gutland then portrays the shift in conscious experience when thinking qualitatively over and above quantitatively.

Masi revisits the current paradigm in consciousness research, i.e., the neurobiological approach that views conscious experience as an epiphenomenal byproduct of neural activity. This material monist theory of consciousness would imply changes in conscious experience if the underlying neurological structures change. Masi, however, reviewed literature on hydrocephalic individuals who have severely diminished neural tissue but preserved mental experience. He thus uses the absence of changes in consciousness to raise questions about the dominant interpretation of consciousness.

Ramminger et al. engage with the methodological and meta-theoretical discourse in neuropsychology. They are considered with the philosophical paradigms underlying research procedures, addressing the controversy between localizationism and holism. Developing a dialogue between these accounts can disclose new assessment methodology for the neuropsychological research on consciousness.

Schleim traces the so-called hard problem of consciousness back to historical precursors in Leibniz and Du Bois-Reymond. This allows him to connect explaining subjective conscious experience with the problem of introspection as Wundt saw it, i.e., that even paying attention to it already alters conscious experience. Schleim then suggests Varela's neurophenomenology and meditation research to conduct consciousness research with an encompassing method and to stabilize conscious experience.

Taguchi and Saigo use category theory to explain a puzzle of time-consciousness: Any given “now” in time is different from the last, yet simultaneously, “now” is always “now.” The flowing and standing now, so they argue, can be captured by the notion of a monoid, while the cosclice category descriptively captures viewing time as consisting of distinct points. In the last part, the authors show how the monoid structure also prevails in meditative states of consciousness.

Wagemann et al. contribute an empirical investigation on the basis of a mixed-methods approach. The subject matter of their investigation is intersubjectivity under the constraint of wearing face masks in the context of the COVID-19 pandemic. Their results support theories of inter-corporeality as they suggest that I-Thou relations unfold in oscillating forms of mental activity that are impeded by social distancing regulations.

Wendler and Fuchs question schizophrenia as a pathological shift in consciousness that leads to utter incomprehensibility and bizarreness. The authors counter that this supposed incomprehensibility's experiential structure can be understood by drawing on phenomenology. They make their case by countering three different sources of confusion: overreliance on delusional beliefs, a false threat of irrationalism, and various equivocations.

Ziegler and Weger use a mathematical example and first-person phenomenology as a guideline to broaden one's attention to the pure thinking action that underlies our daily conscious experience, but that usually goes unnoticed. To stabilize the descriptive particulars of this thinking, the authors contrast its productive and performative nature with various other kinds of thinking, flashes of insight, and mere associating based on memory.

## Author contributions

CG: Conceptualization, Project administration, Validation, Writing—original draft, Writing—review & editing. SK: Conceptualization, Project administration, Validation, Writing—original draft, Writing—review & editing. KL: Conceptualization, Project administration, Validation, Writing—original draft, Writing—review & editing. MM: Conceptualization, Project administration, Validation, Writing—original draft, Writing—review & editing. AW: Investigation, Validation, Writing—original draft, Writing—review & editing, Conceptualization.

